# Disadvantaged Social Groups and the Cigarette Epidemic: Limits of the Diffusion of Innovations Vision

**DOI:** 10.3390/ijerph13121230

**Published:** 2016-12-11

**Authors:** Myriam Khlat, Fred Pampel, Damien Bricard, Stéphane Legleye

**Affiliations:** 1 Institut national d’études démographiques (Ined), 133 boulevard Davout, Paris 75020, France; bricard@irdes.fr; 2 Population Program, University of Colorado, Boulder, CO 80302, USA; fred.pampel@colorado.edu; 3 Institut de recherche et documentation en économie de la santé (Irdes), 117 bis rue Manin, Paris 75019, France; 4 Institut national de la statistique et des études économiques (Insee), 6 Rue Legrand, Malakoff 92240, France; stephane.legleye@insee.fr; 5 Centre de recherche en épidémiologie et santé des populations (CESP), Faculté de médecine—Université Paris-Sud, Faculté de médecine—UVSQ, INSERM, Université Paris-Saclay, Villejuif 94805, France

**Keywords:** cigarette smoking, inequalities, social behavior, addiction, public policy

## Abstract

The original four-stage model of the cigarette epidemic has been extended with diffusion of innovations theory to reflect socio-economic differences in cigarette use. Recently, two revisions of the model have been proposed: (1) separate analysis of the epidemic stages for men and women, in order to improve generalization to developing countries, and; (2) addition of a fifth stage to the smoking epidemic, in order to account for the persistence of smoking in disadvantaged social groups. By developing a cohort perspective spanning a 35-year time period in France and the USA, we uncover distinctive features which challenge the currently held vision on the evolution of smoking inequalities within the framework of the cigarette epidemic. We argue that the reason for which the model may not be fit to the lower educated is that the imitation mechanism underlying the diffusion of innovations works well with regard to adoption of the habit, but is much less relevant with regard to its rejection. Based on those observations, we support the idea that the nature and timing of the epidemic differs enough to treat the stages separately for high and low education groups, and discuss policy implications.

## 1. The Cigarette Epidemic Model: Theory and Revisions

An epidemic model of cigarette use has proven useful in describing patterns of change in smoking prevalence [1]. The model is based on the historical timing of the initial adoption of cigarettes, the early spread among men, the later spread among women, and the subsequent partial reversal among men and, later, the partial reversal among women. It predicts eventual convergence by gender at low levels of smoking in the last stage of the epidemic.

The model has been extended with diffusion of innovations theory [2,3] to include socio-economic position and education differences in cigarette use. Although the original model distinguished only between males and females, the extension to socio-economic position and, in particular, education as its key component, follows the underlying logic of the model. High education groups are the first to adopt cigarettes early in the epidemic, and the first to reject cigarettes later in the epidemic. As cigarette use diffuses throughout the population, low education groups follow high education groups in widely adopting tobacco. Later, the widespread rejection of cigarette use by high education groups occurs first, and is followed by the widespread rejection by others, including low education groups. 

In a recent revision of the model, the developers have noted that there is little regularity in the lag between the timing of the adoption of cigarettes by large numbers of men and adoption by large numbers of women. They call for the separate analysis of the stages of the epidemic for men and women [4]. Further to that, given the limited evidence of convergence in smoking between education groups and continued inequality in smoking-related mortality, Dixon and Banwell [5] suggest adding a fifth stage to the epidemic, characterized by a “sedimentation of smoking in successive low SES (socio-economic status) cohorts”.

Although the latter suggestion is quite sound on descriptive grounds, departure from the theory should lead to a thorough appraisal and adaptation of the foundations of the model. The question of the continued “adoption of a non-innovative and damaging behavior in successive cohorts”, in spite of long-standing tobacco control programs and interventions, has been raised [5]. According to Vedoy [6], the observed patterns suggest that “the diffusion has been blocked or delayed”, and according to Pampel [7], the “stubborn resistance” to anti-smoking measures of low-SEP (socio-economic position) groups may be interpreted as reflecting the “fundamental causes of disease” theory [8]. This issue is of importance as the relevance of the epidemic model to low education groups has profound implications for public health policy. The original model is consistent with a population approach to public health that advocates mass environmental interventions such as raising tobacco prices and restricting public use of tobacco. Comprehensive, broadly-targeted policies are expected to speed the rejection of smoking, compress the timing of the epidemic, and reduce the equilibrium levels of smoking at the end.

## 2. Trends and Patterns

Despite considerable progresses, the picture which emerges raises many questions. Smoking is on the rise in low-income and middle-income countries, and the decline in prevalence has “slowed down to a trickle” in many developed countries [9]. In addition to overall levels, distinct patterns of evolution over time contribute crucially to inequality in mortality and to a critical public health problem. In the last phase of the epidemic, inequalities are expected to initially get stronger, but to taper progressively later on, as the lower educated follow the higher educated in their rejection of cigarette use. During the nineties, smoking inequalities were found to widen across Europe [10], and the tobacco control policies implemented in the 2000s have been accompanied by an accelerated rise in inequalities [11]. This has led to a growing concern over the potential for population-level policies to worsen inequalities in smoking [12].

Growing relative disparities may result from different scenarios. For instance, there may be a slower pace of smoking decline in the lower educated compared to the higher educated, or a stalling in the lower educated as opposed to a regular decline in the higher educated, or a parallel decline or even a rise in the lower educated. Carefully considering those stratum-specific evolutions is essential in order to better understand the dynamics of the smoking epidemic and eventual departures from the diffusion of innovations component of the model. Particularly, knowing whether and when the lower educated “imitate” the higher educated in their rejection of smoking is essential for developing public policies targeting areas of resistance.

## 3. Objectives

In order to further investigate the model along those lines, we examine patterns of change in smoking in different educational categories from successive cohorts. Education is particularly relevant with regard to smoking as it carries the knowledge and skills underlying health behavior choices [13]. For the purpose of attaining a certain degree of generality, we consider two countries, the United States and France, which widely differ in the timing of the cigarette epidemic. Indeed, sales of manufactured cigarettes peaked in 1963 in the United States as opposed to 1985 in France [14]. Given this two-decade lag and the greater maturity of the epidemic in the United States, we may expect the least educated to be much more advanced in their imitation of the rejection of smoking of the higher educated in the United States than in France. The questions are: considering prolonged periods of time, how did the smoking habits of the lower educated and those of the higher educated evolve over cohorts? How did those patterns depart from the expectations defined by the epidemic model? What theoretical and policy implications might follow from divergence of results from the model?

## 4. Methods

Our outcome of interest was smoking prevalence, which is easier to obtain by education levels than smoking-attributed mortality and is a key component of the epidemic model. We compare smoking prevalence by education across years as a rough guide to trends in disparities. In order to provide a more precise indicator of change, we further examine educational differences at the same age for cohorts born during different historical periods and stages of the epidemic. 

Based on cross-sectional survey information on lifetime smoking history, we reconstructed historical data on age-specific smoking prevalence. For this purpose, using age at smoking uptake and age at eventual cessation (which were provided by 99% of the respondents in both countries), we determined the smoking status of each subject by calendar year from birth to year of interview. The two data sets used were (1) the 2010 French Health Barometer, a representative nationwide telephone survey of the non-institutionalized population aged 15–85 years [15]; and (2) the 2010 U.S. National Health Interview Survey Adult Sample, a representative nationwide face-to-face survey of individuals ages 18 and older within households and non-institutional group quarters [16]. For the population studied here, men and women at ages 25–69 years with complete education and smoking data, the sample sizes were 20,940 in France and 20,444 in the United States. In the French survey, information on the highest certificate, diploma, or degree attained was available, with 9 categories ranging from no diploma to university degree. In the United States survey, educational level was categorized into 4 categories ranging from less than high school to bachelor’s degree and higher. As the cohorts experienced diverse educational conditions and the two nations differ in the structure of their educational systems, we defined for comparative purposes a relative measure of education by grouping the most educated (roughly the top 20%) and the least educated (roughly the bottom 20%) for each cohort and sex.

We estimated prevalence of smoking at 30–34 years old by period (from 1970 to 2005), using a 9-year moving average to smooth the estimates. The reason for choosing this age range is that it captures the bulk of smoking uptake that occurs early in life, from the end of adolescence to the early twenties, as well smoking cessation that occurs during early adulthood. By the end of the twenties, those merely experimenting would have quit, and those remaining could be considered as long-term persistent smokers.

## 5. Results

**Figure 1:**
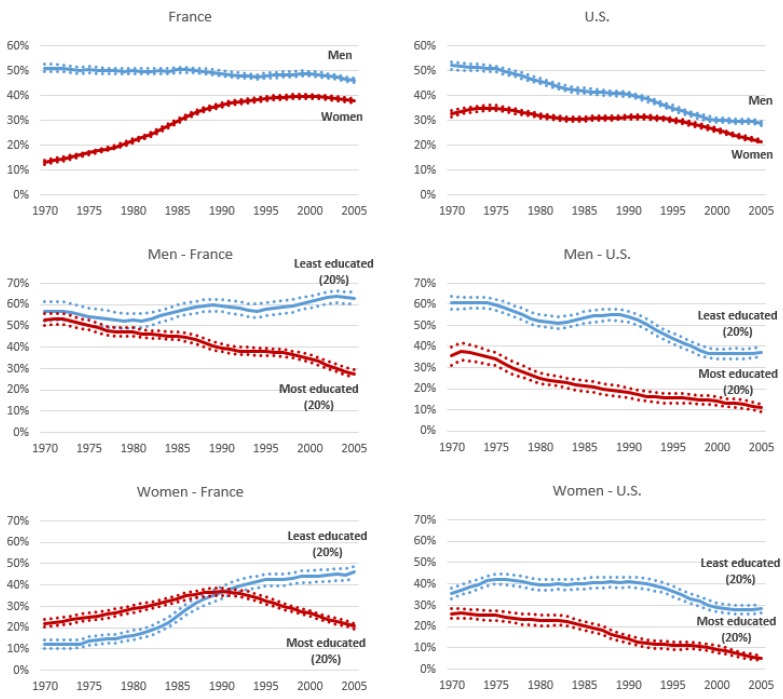
Smoking prevalence in men and women aged 30–34 years from 1970 to 2005, France and the United States (% and 95% confidence intervals). Source: 2010 French Health Barometer, 2010 U.S. National Health Survey Adult Sample.

Figure 1 depicts levels of regular smoking prevalence at ages 30–34 years for men and women in France and the USA, contrasting the top 20% most educated and the bottom 20% least educated. Throughout the observation period, lower levels of smoking are expectedly found in the United States, where the epidemic is more mature, than in France. Furthermore, the relative difference between the most and least educated is rising from the 1990s in both countries and for both genders. The precise patterns underlying this rising trends are, however, very different in the two countries and consistent across genders. 

We observe in the United States a somewhat parallel decline in the two educational groups, with a relatively stable absolute difference, as opposed to a divergent evolution in France, with an enlargement of the absolute differences. Regarding more specifically the least educated, it is worth noting that in the United States, the levels have stalled from 2000 to 2005, after a decade of decline. The most striking feature in this evolution is the smoking rise among the least educated in France, with levels among those aged 30–34 years in 2005 reaching as high as around 60% in men and nearly 50% in women. 

## 6. Discussion

Although the cigarette epidemic model fits the experience of higher education groups well, and perhaps applies to most of the population, it does not represent the experience of all groups. By developing a cohort perspective spanning a 35-year time period, we identify specific and distinctive features that challenge common views of the evolution of smoking inequalities within the framework of the cigarette epidemic. High education groups have already advanced to the final stage of the cigarette epidemic model in many high-income countries, including France and the United States, and those groups fit the timing of the model. Yet, we observe the persistence of high levels of smoking in the lower educated in the United States, and, even more unexpected than that, the pursuit of the rise among those in France, as if those groups were still in a much earlier phase of the epidemic. We show that, while the prevalence among men and women evolve similarly during the past decades, they sharply diverge according to educational level, which supports the idea that education now plays a stronger role than gender as a predictor of smoking. 

We would like to emphasize that the model may be considered as including two cycles of diffusion: the first relates to smoking adoption in the population and the second to smoking rejection, with rejection becoming in turn an innovative behavior in its own right that diffuses based on knowledge on the health effects of tobacco and the progresses made in smoking prevention [17]. As an alternative to Dixon and Banwell [5], who argue in favor of the consideration of a fifth phase in the smoking epidemic, and echoing Thun et al. [4], in their vision of gender-specific epidemics, we propose that the nature and timing of the epidemic differs enough to treat the stages of the epidemic separately for high and low education groups. Such a revision of the model would acknowledge the different mechanisms behind smoking of advantaged and disadvantaged education groups and the changes over time in the profile of the group of smokers. 

The higher educated are likely to quickly experiment and adopt health innovations, and reject innovative habits whenever they are found to be harmful. Such behavior relates to a rationale of experimentation and distinction with limited emotional or addictive components. In contrast, while disadvantaged groups may imitate adoption of cigarette smoking in the early and middle stages of the epidemic, they lack the resources to reject it in the later stages. Rejection requires overcoming an addiction to a substance that helps in coping with the daily difficulties inherent to the life of the less educated groups [18,19]. This accords with the idea that today’s smokers are different from yesterday’s and that they are more addicted, or alternatively that they gain more coping value from the short-term pleasures of smoking, or discount the future more heavily [20]. The continuation of the habit among those groups despite policies to increase the monetary and opportunity costs of smoking, which has been interpreted as a fifth stage in the model [5], can be interpreted as a delay among the lower educated. In either case, the diffusion of innovations theory works well with regard to the cycle of diffusion of smoking adoption, but is much less relevant with regard to the cycle of diffusion of smoking rejection. 

## 7. Conclusions

Inasmuch as tobacco control has been qualified as “the greatest public health success story of the past half century” [9], the persistence of health inequalities, which are related in good part to smoking, is considered as one of the great disappointments of public health [21]. Given the clear harm of tobacco, the divergent trends by education foretell continued inequality in the future mortality and limit progress toward goals for a healthy population. Moreover, the changing life conditions and composition of smokers call for innovation in tobacco control policy [20]. Our vision is consistent with arguments of Frohlich and Potvin [12] that population-based approaches to smoking reduction are limited in the ability to change the behavior of disadvantaged groups and that more inclusive approaches are needed. If women and low education groups deviate from the epidemic model, then policies should consider gender and social groups [22] to better address the special psychological, social and cultural determinants of disadvantaged smokers. Furthermore, the advent of new smoking methods (e-cigarettes and other smokeless products) may be considered as the beginning of a new cycle of diffusion. If, as has been suggested, the rich initiate vaping while the poor pursue smoking [23], we may be facing soon even larger smoking inequalities.
